# Testicular sperm extraction in a patient with ejaculatory dysfunction after combined androgen blockade therapy for prostate cancer

**DOI:** 10.1002/iju5.12176

**Published:** 2020-06-16

**Authors:** Toshiyasu Amano, Chihiro Suzuki, Yuji Shimojima, Tetsuya Imao, Carolyn Earle

**Affiliations:** ^1^ Department of Urology Nagano Red Cross Hospital Nagano Japan; ^2^ Subiaco Sexology Subiaco Western Australia Australia

**Keywords:** combined androgen blockade therapy, ejaculatory dysfunction, prostate cancer, sperm preservation, testicular sperm extraction

## Abstract

**Introduction:**

Prostate cancer is the most prevalent urological cancer for older men. However, there is still a possibility that a few prostate cancer patients may still wish to have children.

**Case presentation:**

A 49‐year‐old male was diagnosed with low‐risk prostate cancer. Combined anti‐androgen blockade therapy was performed for 8 months prior to radiation therapy. However, he suffered from ejaculatory dysfunction and wished to conceive with his partner. Hormonal therapy was discontinued and he was referred to our clinic for sperm preservation. His ejaculatory function did not recover after 4 months discontinuation of hormonal therapy, subsequently micro‐testicular sperm extraction for sperm preservation was successfully performed.

**Conclusion:**

Sperm preservation in patients with prostate cancer is unusual but it should be considered if the patient’s fertility is an issue.

**Abbreviations & Acronyms**
AYA: adolescents and the young adult
CAB: combined anti‐androgen blockade
EjD: ejaculatory dysfunction
LH‐RH: luteinizing hormone releasing hormone
PSA: prostate‐specific antigen
TESE: testicular sperm extraction

**Keynote message**

The subject of this case report was a 49‐year‐old male who was diagnosed with low‐risk prostate cancer. He suffered from EjD after neoadjuvant CAB and wished to conceive with his partner. His ejaculatory function did not recover after 4 months discontinuation of CAB, subsequently micro‐TESE for sperm preservation was successfully performed.

## Introduction

Due to the remarkable progress of protocols for cancer treatment, the number of survivors has increased. Simultaneously, fertility preservation has become a significant issue for those survivors associated with anti‐cancer treatments, especially AYA generation. Prostate cancer is the most prevalent urological cancer for older men. However, there is a possibility that a few prostate cancer patients may still wish to have children. We documented the case of a 49‐year‐old prostate cancer patient with EjD whose sperm preservation was successful after neoadjuvant CAB.

## Case presentation

A 49‐year‐old male with high serum PSA level (4.83 ng/mL) was diagnosed with prostate cancer with Gleason score 4 + 3 in October, 201X. Further examination showed no invasion or metastasis. The physician in charge recommended performing radical treatment with surgical intervention or radiation. The patient decided to be treated with radiation combined with neoadjuvant hormonal therapy. CAB along with oral intake of anti‐androgen medicine and LH‐RH analogue injection were instigated from March, 201X + 1. CAB was continued for 8 months and the serum PSA level reduced to <0.01 ng/mL. However, he suffered from EjD and still wanted to procreate in the future. Therefore, the LH‐RH analogue injection and oral intake of anti‐androgen medicine was discontinued in October and November, 201X + 1, respectively. He was transferred to our clinic for the purpose of sperm preservation in February, 201X + 2.

His physical examination revealed normal bilateral testes size (15 mL), normal vas deference and absence of varicoceles. The results of his biochemistry tests showed luteinizing hormone 0.19 mIU/mL, follicular stimulating hormone 2.85 mIU/mL, prolactine 6.39 ng/mL, total testosterone 0.10 ng/mL, and PSA 0.02 ng/mL His genotype was 46, XY and partial defect of Y gene (AZFc, gr/gr) was observed.

**Fig. 1:**
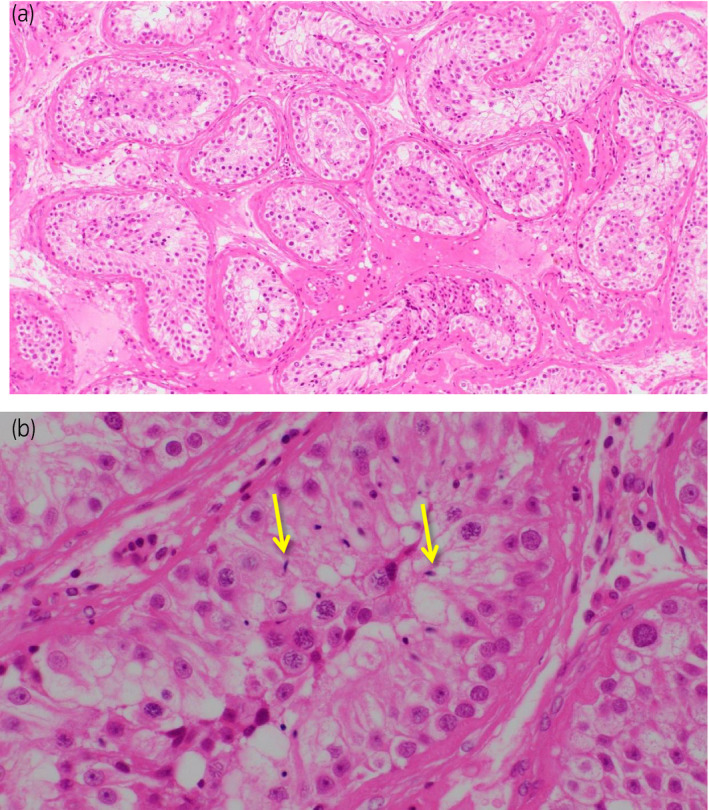
Histological findings of seminiferous tubules. (a) H & E, ×100. Mild to moderate decrease in spermatogenesis were observed. Thicknesses of basement membrane of sperm tubules were widely found. (b) H & E, ×400. A few sperm were discovered (arrows), and sperm preservation was performed from several parts of testicular tissues.

Since a cycle of spermatogenesis takes approximately 75 days, CAB was discontinued for 4 months. However his EjD did not recover. Subsequently, left micro‐TESE for sperm preservation was performed in March, 201X + 2. Most of the seminiferous tubules were meandering, but were thin. A few spermatozoa with low motility were observed and estimated as Johnsen’s score count 8 (Fig. 1). Thus several seminiferous tubules were removed to obtain sufficient spermatozoa to freeze. Finally, 10 sperm storage tubes each containing 50 to 200 spermatozoa were frozen. There were no post‐operative complications.

After 2 months CAB from April, 201X + 2, he had received a total of 78 Gy radiation at a local hospital. His spermatozoa had been preserved until they would be required for fertility treatment.

## Discussion

Due to the advancement of treatment and early detection of cancer, there has been an increase in survival rate and improvements in the quality of life of these patients. However, preservation of fertility is an important issue especially for AYA generations. Sperm preservation is a significant consideration for many patients with testicular tumors, leukemia, or malignant lymphoma.^1^ Currently in Japan, the mean age of marriage in both men and women is rising. Hence, the age when couples consider having a family is also increasing.^2^ Therefore, it is important to consider fertility issues in not only the AYA generation but also older patients who are being treated for their cancer.

Prostate cancer was reported as one of the most prevalent malignant neoplasms in older men.^3^ Strategies for treatment of prostate cancer include surgical, radiation, and hormonal therapy according to their stage and the patient’s clinical history. After surgical or radiation therapy for localized prostate cancer, almost all the patients suffer from erectile dysfunction and EjD.^4^ It was reported that 3.7% of patients responded that infertility was a side effect of prostate cancer treatment which caused them the most concern.^5^ One out of five prostate cancer patients were reported to bank sperm before radical prostatectomy in Italy.^6^ Of 122 men with cancer who requested semen cryopreservation in Japan, eight patients with prostate cancer were included.^7^ Thus, patients with localized prostate cancer who have indications for surgical intervention or radiation therapy should be offered the opportunity to maintain their fertility via sperm preservation.

Hormonal therapy with CAB is our treatment of choice for older or advanced cancer patients. Neoadjuvant endocrine treatment also has its place in the external beam radiotherapy.^8^ However, CAB induces decreased libido and impairment of spermatogenesis.^4^ Fertility issues are generally not as important to the patients who have a preference for CAB therapy. Although only a few case reports of successful sperm extraction after hormonal therapy have been published, Wood *et al.* reported that the patient had EjD which is a significant problem of male fertility.^9^

This case revealed a partial defect of Y gene (AZFc, gr/gr), which is not a significant indicator for male infertility. Whilst receiving neoadjuvant CAB for 8 months, he decided to pursue sperm preservation. Since a cycle of spermatogenesis takes approximately 74 days,^10^ the efficacy of treatments for spermatogenesis should begin at 74 days after or more. Although the best timing to obtain high quality sperm after CAB is not clear, we expected recovery of spermatogenesis after 4 months discontinuation of CAB. However, he has still suffered from EjD. In addition, it was considered to be difficult to achieve sperm preservation by conventional TESE because spermatogenesis may be damaged due to CAB. Thus, we performed micro‐TESE and obtained a successful sample for freezing.

As the patient had already started CAB therapy before micro‐TESE, sperm extraction from testis tissue after castration was considered to be one of the best methods for both sperm preservation and hormonal prostate cancer therapy. However, he declined castration. Although his EjD continued, this case report does reveal that sperm preservation by micro‐TESE can be possible in prostate cancer patients after a period of CAB discontinuation,

## Conclusion

Sperm preservation by micro‐TESE is a useful procedure in prostate cancer patients with hypo‐spermatogenesis and EjD after CAB.

## Conflict of interest

The authors declare no conflict of interest.

## Ethical statement

The protocol for the research project including human subjects has been approved by a suitably constituted Ethics Committee.
